# Validation of automated positive cell and region detection of immunohistochemically stained laryngeal tumor tissue using digital image analysis

**DOI:** 10.1016/j.jpi.2023.100198

**Published:** 2023-01-29

**Authors:** Hilde J.G. Smits, Justin E. Swartz, Marielle E.P. Philippens, Remco de Bree, Johannes H.A.M. Kaanders, Sjors A. Koppes, Gerben E. Breimer, Stefan M. Willems

**Affiliations:** a Department of Radiotherapy, University Medical Center Utrecht, Utrecht, the Netherlands; b Department of Otorhinolaryngology – Head and Neck Surgery, University Medical Center Utrecht, Utrecht, the Netherlands; c Department of Head and Neck Surgical Oncology, University Medical Center Utrecht, Utrecht, the Netherlands; d Department of Radiation Oncology, Radboud University Medical Center, Nijmegen, the Netherlands; e Department of Pathology, Erasmus University Medical Center, Rotterdam, the Netherlands; f Department of Pathology, University Medical Center Utrecht, Utrecht, the Netherlands; g Department of Pathology and Medical Biology, University Medical Center Groningen, Groningen, the Netherlands

**Keywords:** Computational pathology, Positive cell detection, Positive region detection, Validation, Biomarker analysis

## Abstract

**Objectives:**

This study aimed to validate a digital image analysis (DIA) workflow for automatic positive cell detection and positive region delineation for immunohistochemical hypoxia markers with a nuclear (hypoxia-inducible factor 1α [HIF-1α]) and a cytoplasmic (pimonidazole [PIMO]) staining pattern.

**Materials and methods:**

101 tissue fragments from 44 laryngeal tumor biopsies were immunohistochemically stained for HIF-1α and PIMO. QuPath was used to determine the percentage of positive cells and to delineate positive regions automatically. For HIF-1α, only cells with strong staining were considered positive. Three dedicated head and neck pathologists scored the percentage of positive cells using three categories (0: <1%; 1: 1%–33%; 2: >33%;). The pathologists also delineated the positive regions on 14 corresponding PIMO and HIF-1α-stained fragments. The consensus between observers was used as the reference standard and was compared to the automatic delineation.

**Results:**

Agreement between categorical positivity scores was 76.2% and 65.4% for PIMO and HIF-1α, respectively. In all cases of disagreement in HIF-1α fragments, the DIA underestimated the percentage of positive cells. As for the region detection, the DIA correctly detected most positive regions on PIMO fragments (false positive area=3.1%, false negative area=0.7%). In HIF-1α, the DIA missed some positive regions (false positive area=1.3%, false negative area=9.7%).

**Conclusions:**

Positive cell and region detection on biopsy material is feasible, but further optimization is needed before unsupervised use. Validation at varying DAB staining intensities is hampered by lack of reliability of the gold standard (i.e., visual human interpretation). Nevertheless, the DIA method has the potential to be used as a tool to assist pathologists in the analysis of IHC staining.

## Introduction

Immunohistochemistry (IHC) is widely used for diagnostic and prognostic purposes to determine the expression of various proteins (biomarkers) in tissue samples. Pathologists often evaluate IHC-stained tissue samples and manually evaluate biomarker expression and staining patterns. Since this process is laborious and time-consuming, it can limit the scale of research.^1^ Moreover, visual inspection is inherently semi-quantitative and can introduce subjectivity. A need exists for standardized and preferably automated quantification of IHC measurements to overcome these obstacles.

Recent advancements in digital image analysis (DIA) offer the potential to overcome some of these drawbacks.^1^ Qupath is an open-source bioimage analysis software with the functionality to evaluate biomarkers in digitized histopathological tissue sections.^2^ QuPath is designed to handle whole slide images and it offers the ability to determine the presence of biomarkers and assess their distribution across tumor tissue.

Recently, we developed a DIA workflow to compare the DAB staining patterns of two hypoxia biomarkers, pimonidazole (PIMO), and hypoxia-inducible factor 1-alpha (HIF-1α), which we validate in de present study.^3^ In this workflow, a cell-based analysis was used, determining the DAB positivity per cell instead of counting the number of positive pixels, as was done in previous studies.^4^^,^^5^ This current approach has the advantage of only taking into account staining in the relevant cell region, which makes it easier to compare nuclear to cytoplasmic biomarkers. While the study found only a weak correlation between the hypoxia markers, it showed the feasibility of a DIA workflow to detect positive DAB-stained cells and regions. However, before implementation in the clinic, both the automated quantification of DAB staining, as well as the positive region detection need to be validated.

In this paper, we set out to validate Qupath’s performance for positive cell detection and positive region detection for PIMO and HIF-1α IHC by comparing the results of a DIA workflow to pathologists’ visual evaluation.

## Methods

### Material

Laryngeal tumor biopsies from 58 patients with advanced laryngeal squamous cell carcinoma were used in this study. The patients participated in a phase III randomized trial where they received accelerated radiotherapy with or without carbogen gas and nicotinamide.^6^ 79 patients participated in a multicenter translational side study and received PIMO intravenously two  hours before biopsy. In this present study, the tissue of 58 patients who were included in the RadboudUMC, Nijmegen, the Netherlands (single-center) was used.

Consecutive sections were cut from each tissue block and immunohistochemically stained for two hypoxic markers, HIF-1α, and PIMO. The staining procedure was done as previously described.^3^ For PIMO, we used the primary antibody Mouse-antiPIMO (Lot# 9.7.11, HydroxyProbe, Massachusets, USA). For the HIF-1α IHC, the Novolink kit (Leica Biosystems, Rijswijk, the Netherlands) was used with the primary antibody Mouse-anti-HIF-1α (BD Biosciences, cat# 610959, lot 4 073 775).

### Digital analysis

**Fig. 1:**
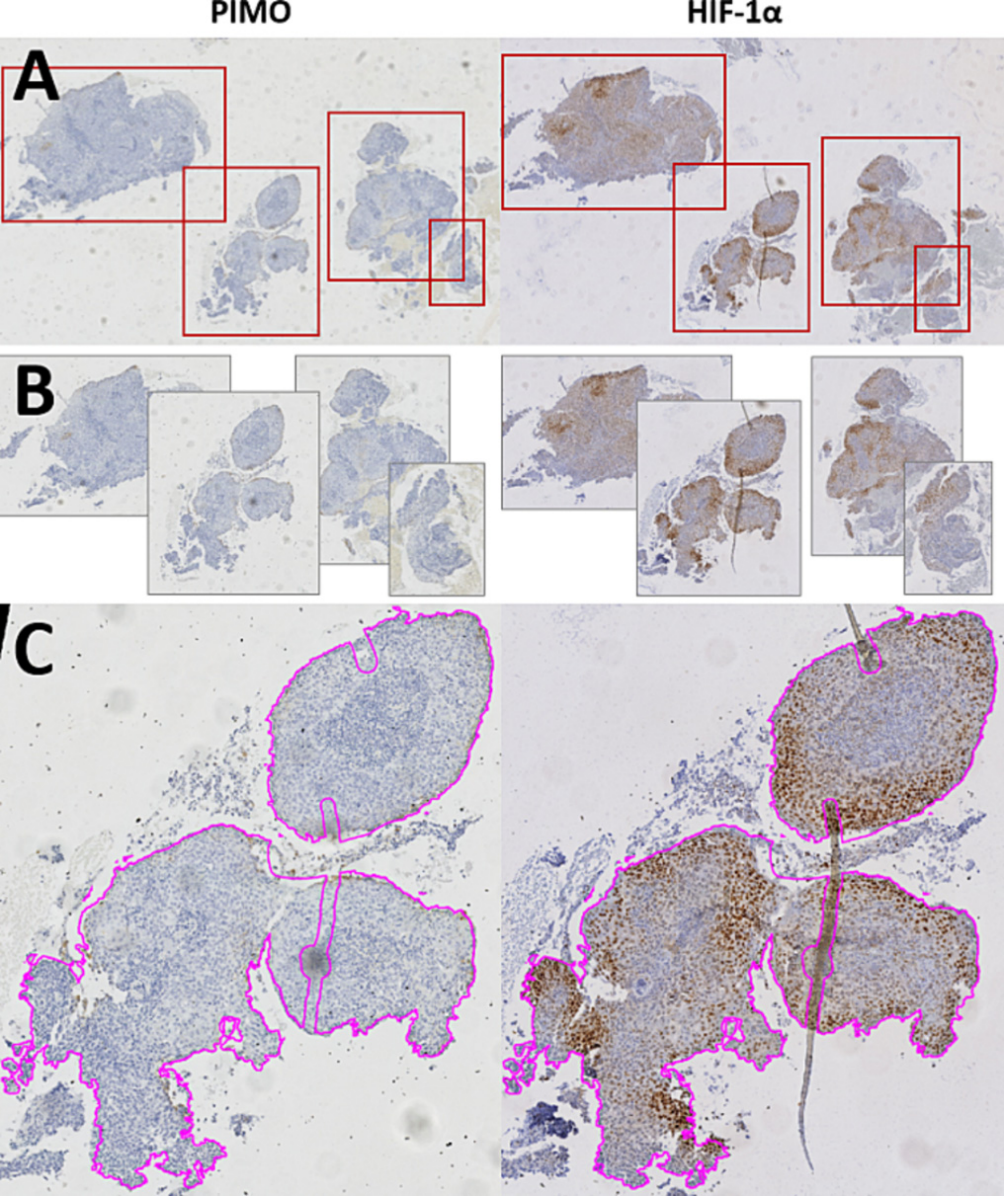
Image preprocessing workflow of PIMO (left) and HIF-1α (right) stained laryngeal tumor biopsies. Isolated tissue fragments are automatically detected (A) and exported into separate image files (B). Corresponding PIMO and HIF-1α fragments are registered to each other (C) and an automatic tissue detection is performed (magenta). Artifacts are manually removed from the annotation.

A Hamamatsu NanoZoomer XR scanner was used for digitizing all sections at 40x magnification. If sections contained multiple tissue fragments, each fragment was exported as a separate image file, Fig. 1. The corresponding PIMO and HIF-1α fragments were manually registered. Tissue fragments that contained no tumor cells or were deemed to be of poor quality due to scanning or tissue artifacts were excluded. A total of 101 matching tissue fragments could be identified in 44 patients (1–9 fragments per patient) and were included in the study. Tissue detection was used to automatically annotate each tissue fragment. Artifacts like folded tissue or dust were manually removed from the annotation. The overlapping area of the annotations in the registered HIF-1α and PIMO tissue fragments was used for analysis.

Positive cell detection was performed in QuPath version 0.3.0 using the settings that can be found in Supplementary material S1. QuPath’s cell detection is based on nucleus detection, which is isotropically expanded to determine the cytoplasm. The two biomarkers showed distinct staining patterns: HIF-1α staining was quite strong and diffuse throughout the tissue, while positive PIMO staining was much weaker. The original study hypothesized that areas of intense HIF-1α staining corresponded to areas of any PIMO staining.^3^ In order to test this hypothesis, only cells with strong HIF-1α intensity were considered positive. The threshold was determined by creating a composite training image of 17 tissue fragments from different patients. On this training image, the best threshold for strong HIF-1α staining was manually identified by a researcher (J.E.S.) who was blinded to the PIMO staining in corresponding tissue fragments.

Cells with strong HIF-1α staining were defined as cells with an optical density (OD) of the DAB color ≥0.65 in the nucleus. Because the staining intensity of PIMO was relatively weak, the threshold for PIMO positivity was set at DAB OD ≥0.10 in the cytoplasm. After classification of all detected cells, the area percentage of positive cells was calculated for each fragment as the area of all positive cells divided by the area of all cells.

**Fig. 2:**
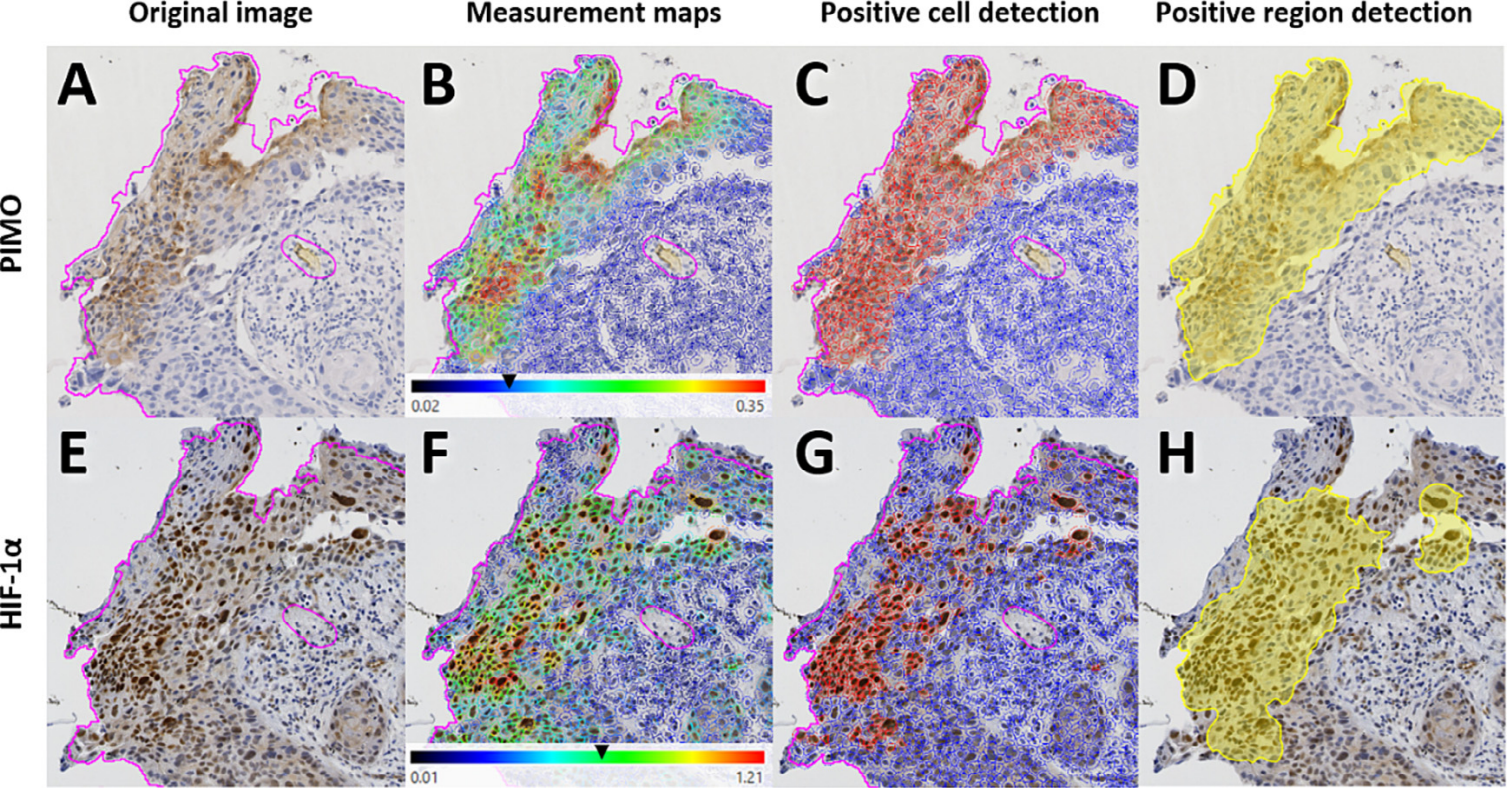
Positive cell and region detection for PIMO (top row) and HIF-1α (bottom row). Cell detection is performed on the original image (A,E) within the tissue annotation (magenta). The optical density of the DAB-staining is measured in each cell’s cytoplasm for PIMO and nucleus for HIF-1α fragments. The black arrows in the measurement maps (B,F) show the threshold for positivity that is used to separate positive (red) from negative cells (blue) (C,G). Areas with a high density of positive cells form positive regions (yellow) (D,H).

After positive cell detection, positive regions were automatically annotated, Fig. 2. A publically available script was used^7^ to annotate regions with a high density of positive cells using the settings in Supplementary material S1.

### Validation

This study validated two aspects of the DIA workflow: positive cell detection and delineation of positive regions. This was done by comparing the results of the DIA to the manual scoring of three experienced head and neck pathologists (S.M.W., S.A.K., and G.E.B.). All observers were blinded to the results of the DIA and to the results of other observers.

#### Positive cell detection

All observers were asked to score the area percentage of positive cells in each tissue fragment. For PIMO, observers were instructed to only assess cytoplasmic staining. For HIF-1α, observers were instructed to only assess nuclear staining and to regard only cells with a strong nuclear DAB intensity as positive. Strong intensity HIF-1α staining was determined beforehand in a consensus meeting with all three observers where examples of strong staining were delineated and agreed upon. A semi-quantitative scoring method was used with three categories (score 0: <1% tissue area was positive, score 1: 1%–33% positivity, score 2: >33% positivity). Each observer scored two-thirds of all fragments in such a way that each fragment was scored by two observers. Disagreements between observers were solved in a consensus meeting with all three observers. In this meeting, observers were blinded to the original scores. The observers’ score through consensus is considered the reference standard.

#### Positive region delineation

The three pathologists were also asked to delineate positively stained regions on a subsection of fragments. For PIMO fragments (n = 14), this meant areas with any staining, while for HIF-1α fragments (n=14) only strongly stained areas had to be delineated. If the DIA detected a positive region that was not delineated by any observer, this region was seen as a false positive. Inversely, if the DIA failed to detect a positive region that was delineated by at least two observers, the region was seen as a false negative. The selection of fragments for this analysis included four PIMO and three HIF1-α fragments in which the DIA did not identify any hotspots.

### Statistical analysis

#### Positive cell detection

The interobserver agreement between pathologists before the consensus meeting was calculated, as well as the linearly weighted Cohen’s kappa for PIMO and HIF-1α fragments separately. The Cohen’s kappa expresses the agreement corrected for agreement by chance. To validate the DIA, the observers’ consensus score and the DIA categorical score were compared by calculating the percentage of agreement and the weighted kappa.

#### Positive region delineation

The accuracy of the DIA hotspot delineation was tested by determining the percentage of false positive and false negative areas. Additionally, three parameters were calculated to quantify the overlap between the DIA hotspots and the true positive areas. The positive predictive value (PPV) determines the probability of the DIA delineated area being truly positive and is calculated as follows: *TP*/(*TP* + *FP*). The sensitivity determines the probability of the true positives being included in the DIA delineation: *TP*/(*TP* + *FN*). The DICE score calculates the overlap between the two areas: *TP*/(2 ∗ *TP* + *FP* + *FN*).

## Results

### Interobserver agreement

Before the consensus meeting, observers agreed on the positivity score in 65.4% of PIMO and 62.4% of HIF-1α fragments, achieving a weighted kappa of 0.43 and 0.33 for PIMO and HIF-1α, respectively. All cases of disagreement between observers were differences of one category and were solved in a consensus meeting.

### Positive cell detection

**Fig. 3:**
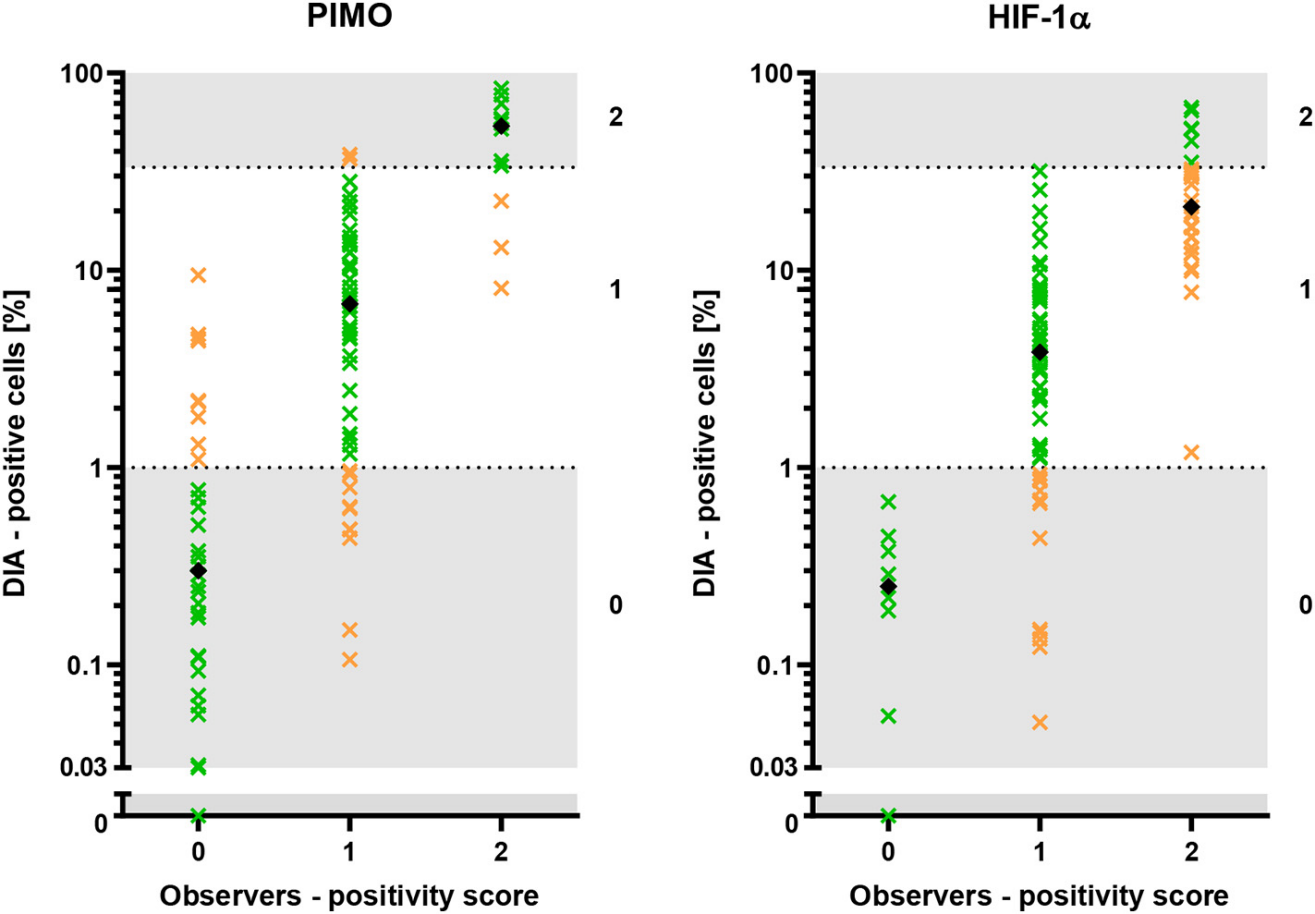
Percentage of positive cells as calculated by the DIA (logarithmic scale) versus scored categorically by observers for PIMO (left) and HIF-1α fragments (right). Observers used a semi-quantitative scoring method (0: <1%, 1: 1%–33%, 2: >33%) depicted as horizontal stripes in the graph. Green points represent a categorical agreement between the DIA and observers, orange points a disagreement of one category. Back diamonds indicate the median DIA positivity for each category. Zero was artificially added on the y-axis, as six PIMO fragments and one HIF-1α fragment contained no positive cells according to the DIA.

The results of the cell detection validation are shown in Fig. 3, where the DIA percentage of positive cells was compared to the observers’ consensus.

For PIMO fragments, the DIA categorical positivity score was in agreement with observers in 77 fragments (76.2%, weighted kappa=0.64). All disagreements were a difference of one category. In 13 of 24 cases of disagreement (54.2%), the DIA gave a higher score than the observers. Each category’s median DIA percentage was within the range of that category.

In the HIF-1α fragments, the DIA agreed with observers in 66 fragments (65.4%, weighted kappa=0.38). All disagreements were a difference of one category. In all cases of disagreement, the DIA gave a lower score than the observers, underestimating the positivity. To the 29 fragments that the observers scored as 2 (>33% positive cells), the DIA gave a median percentage of positivity of 21.0%, giving 23 fragments a score of 1.

### Positive region delineation

Of the 14 PIMO fragments analyzed, there were nine fragments in which both the observers and the DIA delineated positive regions. In these fragments, the DIA resulted in an average PPV of 43.5%, sensitivity of 85.6%, and DICE score of 57.1% when compared to the true positive regions (regions delineated by at least two observers). Four PIMO fragments were correctly identified as negative by the DIA as they contained no true positive regions. This leaves one fragment where the DIA falsely delineated positive regions. The PPV and DICE score of this fragment were 0, and the sensitivity could not be determined.

Of the 14 HIF-1α fragments, 11 contained positive regions according to both the DIA and observers. In these fragments, the DIA had an average PPV of 80.3%, sensitivity of 61.1%, and DICE score of 65.0% when compared to the true positive regions. The three remaining HIF-1α fragments did not contain positive regions according to the DIA, but true positive regions were delineated by observers. Because the DIA identified these fragments as negative, the sensitivity and the DICE score of these fragments were 0, and the PPV could not be determined.

### Patterns of classification

**Fig. 4:**
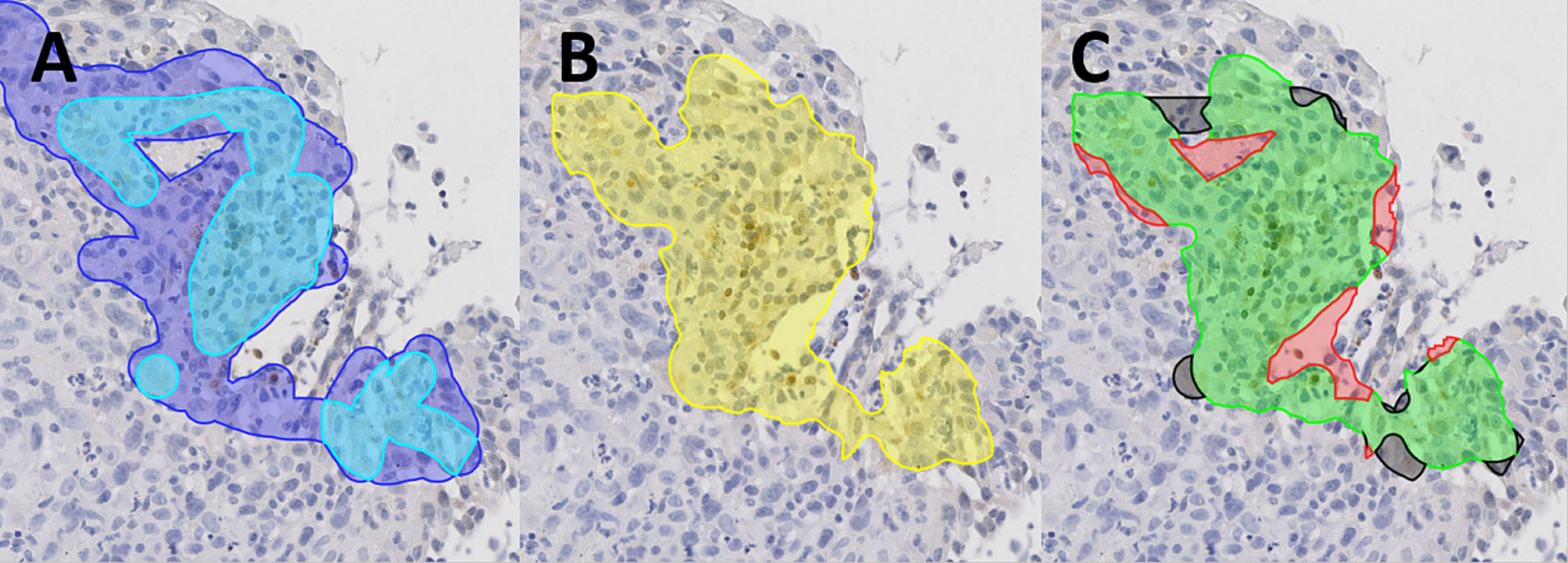
Example of positive region detection result where misclassifications are due to small differences in delineation. The observer delineations (A), divided into areas delineated by one observer (blue) and at least two observers (cyan), are compared to the DIA delineation (B) (yellow). The results (C) show the correctly identified regions (green) and misclassifications. False positives (red) are the regions delineated by the DIA, but not by any observer. False negatives (black) are the regions delineated by at least two observers, but not by the DIA.

When looking at regions instead of fragments, most false positive and false negative regions were due to small differences in the exact delineation of a region, Fig. 4. However, we identified distinct patterns in the classification results that were unique for PIMO and HIF-1α fragments.

**Fig. 5:**
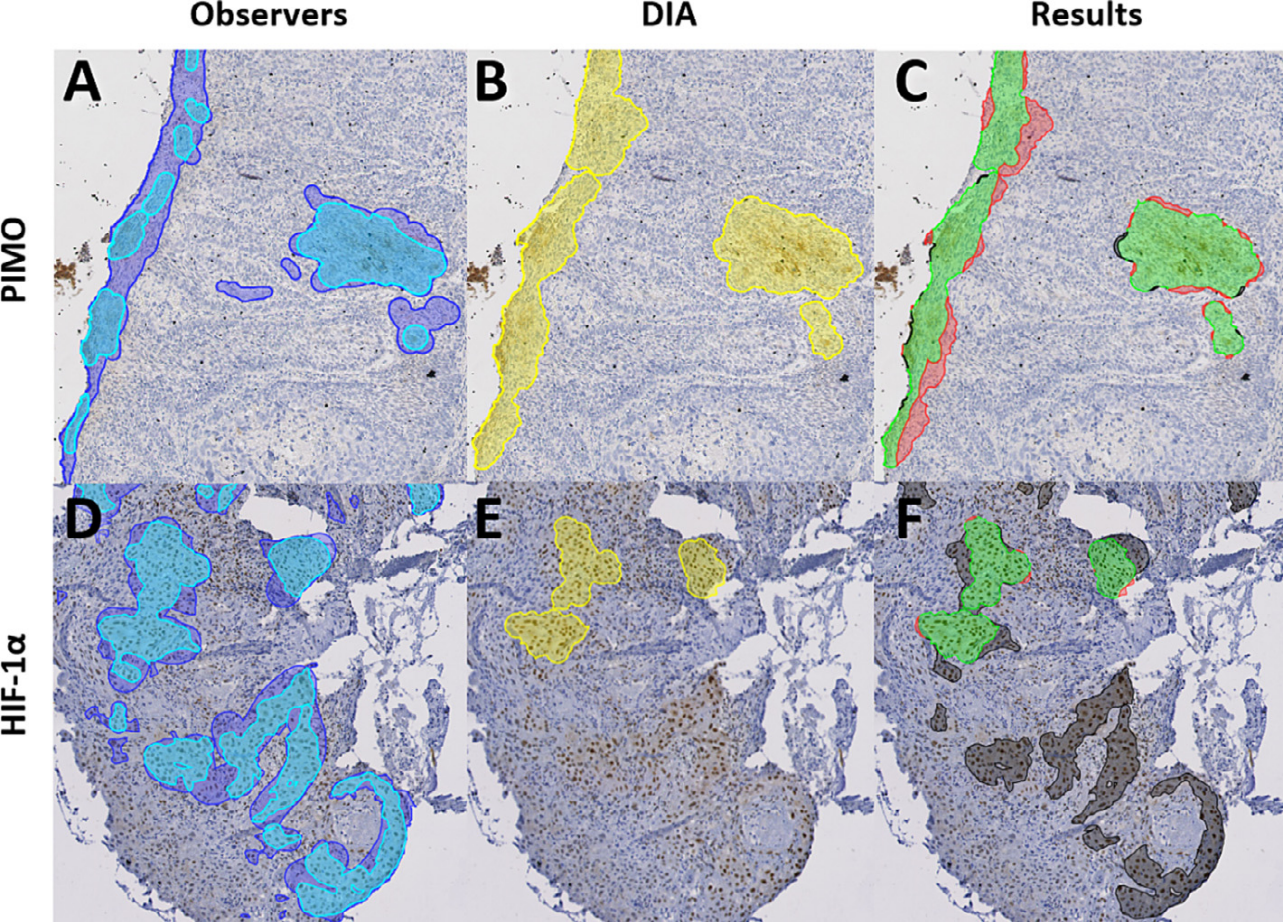
Patterns of classification results on PIMO (top row) and HIF-1α fragments (bottom row). The observer delineations (A,D), divided into areas delineated by one observer (blue) and at least two observers (cyan), are compared to the DIA delineation (B,E) (yellow). The results (C,F) show the correctly identified regions (green), false positives (red) and false negatives (black).

In PIMO fragments, the DIA falsely classified 2.4% of the tissue area as positive and only 0.7% as false negative. While the DIA detected most positive regions, it created slightly wider margins for those regions than the observers did. An example of this pattern is shown in the top row of Fig. 5.

In HIF-1α fragments, the DIA classified 1.3% of the tissue area as false positives and 9.7% as false negatives. This high false negative rate was not only due to differences in margin but also by true positive regions missed by the DIA, Fig. 5.

**Fig. 6:**
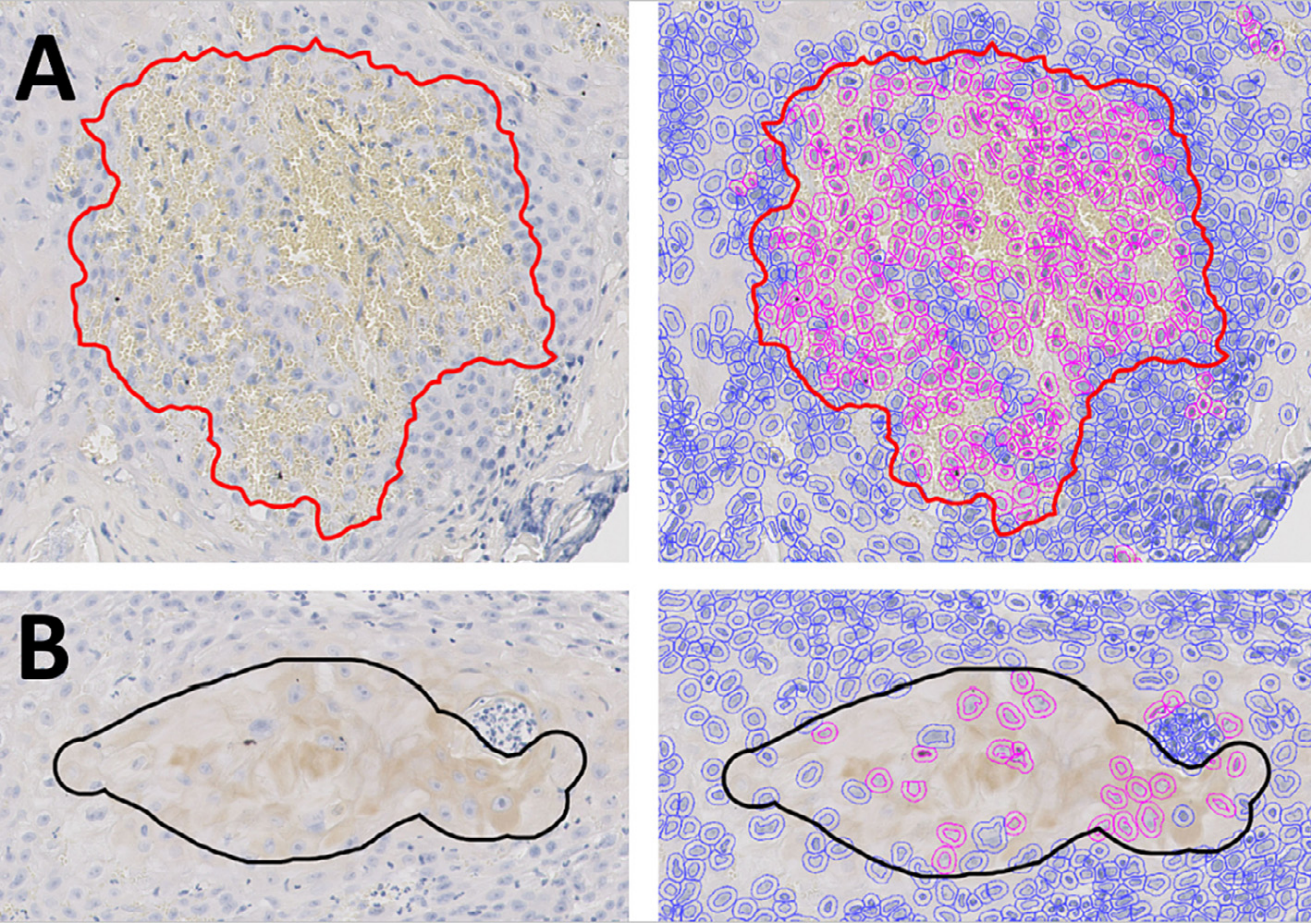
Examples of false positives (red) due to red blood cells (A), and false negatives (black) due to low cell density (B). On the left, the original tissue is shown with the delineated areas, on the right the positive (pink) and negative (blue) cell detections are shown.

Additionally, we identified two specific situations in which misclassification occurred. The first is false positives due to red blood cells, Fig. 6A. Because the PIMO threshold is relatively low, the small amount of brown pigment in the red blood cells was picked up by the DIA workflow as positive staining. The second type of misclassification are false negatives due to low (detected) cell density. Positive regions are delineated based on a high density of positive cells. If the detected cell density is too low, it will not annotate the region, even if the detected cells are positive, Fig. 6B.

## Discussion

In this paper, we set out to validate a method that automatically detects positive IHC stained cells and regions in tissue biopsies. This was done by comparing the results of the DIA and pathologists in determining the percentage of positive cells and the location of positive regions.

The DIA achieved a weighted kappa of 0.64 for PIMO and 0.38 for HIF-1α fragments, corresponding to a substantial and fair agreement, respectively.^8^ In HIF-1α fragments, the DIA tended to underestimate the amount of positivity compared to the observers’ scores.

The agreement between the DIA and observers is comparable to the interobserver agreement between pathologists before consensus was reached in HIF-1α fragments (weighted kappa of 0.33). In PIMO fragments, the DIA’s results were better than the initial interobserver agreement (weighted kappa of 0.43). This suggests that the performance of the DIA in determining the percentage of positive cells is comparable to a pathologist’s visual estimation.

When validating the region detection, we found that the DIA tended to slightly overestimate the PIMO positive regions, while missing positive HIF-1α regions. In PIMO fragments, the DIA falsely classified 2.4% of the tissue area as positive. These false positive regions were predominantly due to wider margins created by the DIA around positive regions. Since this still resulted in a correct detection of positive regions, we find this result acceptable.

In HIF-1α however, the DIA falsely classified 9.7% of the tissue as negative, missing several regions that observers annotated as positive. For clinical use, this result is unacceptable.

For HIF-1α fragments, a relative positivity threshold was used, so only strong DAB intensity was considered positive. For such a threshold, a DIA workflow might outperform observers. Humans can easily recognize relative intensity differences within one image, but find it much harder to determine absolute staining intensity when looking at fragments in isolation.^9^ Observers might delineate areas with the highest staining intensity within one fragment, even when the absolute staining intensity is relatively low compared to other fragments. Doing so introduces the risk of overestimating a fragment’s positivity, resulting in more false negatives. The DIA workflow might thus be better equipped to consistently compare the staining intensity across different images.

In our present study, it is unclear whether the high false negative rate in HIF-1α fragments is due to poor performance of the DIA or overestimation of positive region by observers. This makes it difficult to draw any conclusions regarding the validity of the DIA method when it comes to detecting strong DAB staining.

This brings us to the gold standard paradox^9^: how do we validate a method when the reference standard is not an objective measure? In the current study, we tried to minimize the effect of interobserver variability by only taking into account the consensus of all observers. However, before consensus, observers in this study only agreed on the positivity score in 63.9% of cases. This underlines the challenges of visual assessment, as pathologists remain subjective to visual and cognitive biases when it comes to assessing color, intensity, and contrast.^9^ On the other hand, trained pathologists can better discern non-specific staining from clinically relevant staining, an ability that algorithms lack. For example, the PIMO staining in this study would often be located around the edges of the tissue. This so-called “edge effect” occurs when tissue lifts from the slide, causing both sides of the tissue to be exposed to the antibodies as well as making it more difficult to wash off excessive antibodies.^10^ While a pathologist would recognize this as non-specific staining due to the location and pattern of the stain, the DIA workflow simply recognizes positive cells. Such areas in the tissue should either be manually removed from the annotation before analysis or more refined AI solutions need to applied that filters out these false positives.

### Digital image analysis workflow

This study shows that digital, cell-based analysis of IHC stains is feasible on biopsy tissue. Previous studies have mainly focused on Tissue Micro-Arrays (TMAs) material or specific regions of interest,^11^, ^12^, ^13^, ^14^ but clinical practice asks for robust methods to process whole-slide tissue biopsies. Biopsy tissue contains more artifacts and non-tumorous tissue than TMAs and also deals with different sizes of tissue area and variation in IHC batches. Methods that can accurately assess the positive fraction of biopsy material can streamline the pathology workflow in clinical practice and propel the scale of research. It also overcomes the common problem of interobserver variability in assessing the positive fraction of IHC stains.^15^^,^^16^

A cell-based analysis has the advantage of only considering staining positivity in the relevant cell compartment, e.g., DAB staining of nuclear markers is only measured in cell nuclei. This diminishes the influence of non-specific staining. However, this method relies on a robust cell detection. QuPath’s cell detection functionality, while performing well in general, could be further improved, e.g., for separating connecting cell nuclei. To address issues like this, QuPath continually improves its functionality and adds extensions for the application of different cell detection methods like StarDist.^17^

When applying the method to other data or when using it for other IHC markers, it is advised to work closely with experienced pathologists when determining positivity thresholds. However, using an absolute intensity threshold makes the method vulnerable to intensity inhomogeneity, artifacts, noise, and interbatch variability in DAB intensity. Therefore, color normalization of the DAB staining should ideally be performed before analysis to overcome these issues.^18^

Another improvement of this methodology could be to include an object classifier that can separate tumor cells from stromal tissue, immune cells, or artifacts. A similar study by Rizzardi et al. (2012) implemented a tumor–stroma classifier to determine S100A1 positivity on TMA material and found high correlations between DIA-estimated DAB positivity and semi-quantitative scoring by pathologists.^11^

In the current workflow, all scanning and tissue artifacts are manually removed from the analyzed annotations. Several methods have been developed to automatically detect artifacts like dust, tissue folds, and out-of-focus scans.^19^^,^^20^ Implementation of such methods in the existing workflow could save time and increase the throughput time of tissue.

As it stands right now, the workflow needs further improvement before unsupervised positive cell detection is feasible. However, it can serve as a tool to assist pathologists when assessing IHC sections. The availability of computer-aided measurements decreases interobserver variability when determining IHC positive fractions.^21^^,^^22^ Therefore, the main application of the method would be to use its results as a reference point, which pathologists can adjust based on context or perceived misclassifications. Decreased interobserver variability would be of great value in translational biomarker research, where the method could be used to achieve a more robust evaluation of the presence and location of biomarkers.

### Limitations

As mentioned before, a major limitation of this study is the use of a relative threshold for HIF-1α positivity. The gold standard of visual assessment by observers was not sufficiently reliable when it comes to assessing staining intensity. This makes it difficult to determine whether discrepancies between the two methods are due to poor performance of the DIA or mistakes in the observers’ assessment. Therefore, we cannot draw any conclusions regarding the DIA’s performance in detecting strong HIF-1α positivity.

The Cohen’s kappa is designed to account for the agreement by chance. However, the (weighted) kappa has a known limitation when there is a skewed distribution of the data, which is the case in our dataset.^23^ This will negatively affect the value of the kappa and makes it difficult to compare the kappa’s between different datasets.

The display screens used by the different observers were not color calibrated. Color settings, as well as brightness and contrast of the display, can influence the hue of stained cells, which might influence a pathologist’s evaluation of the tissue. However, previous studies have concluded that monitor settings and quality had little to no impact on pathologists’ assessment of digital pathology slides.^24^

### Conclusions

We have shown that positive cell and region detection on biopsy material is feasible, but in need of further optimization before unsupervised use. Validation of the DIA workflow for detection of strong DAB staining was hampered, because the gold standard (visual assessment) is not sufficiently reliable in assessing staining intensity. Nevertheless, the DIA method has the potential to be used as a tool to assist pathologists in the analysis of IHC and to decrease interobserver variability in translational biomarker research.

## Funding

The original Phase III trial was supported by the 10.13039/501100004622Dutch Cancer Society (KWF) Research Fund No. CKTO-2000-09 and KUN-2008-4088 and a European Community Grant No. FP7-HEALTH-2007-B222741, project METOXIA (Metastatic Tumors Facilitated by Hypoxic Tumor Micro-Environments). No additional funding was received for the present study.

## Declaration of interests

The authors declare that they have no known competing financial interests or personal relationships that could have appeared to influence the work reported in this paper.

## Appendix A Supplementary data

Supplementary material Image 1
