# Supplementary material for: Validation of automated positive cell and region detection of immunohistochemically stained laryngeal tumor tissue using digital image analysis
Source: J Pathol Inform. 2023 Jan 29;14:100198. doi: 10.1016/j.jpi.2023.100198 (PMC9930147; doi:10.1016/j.jpi.2023.100198)
Supplement: Supplementary file 1 — Supplementary material [file mmc1.docx]

# Supplementary Material S1

**Cell detection settings**

| HIF-1α staining | runPlugin('qupath.imagej.detect.cells.PositiveCellDetection', '{"detectionImageBrightfield": "Optical density sum", "requestedPixelSizeMicrons": 0.5, "backgroundRadiusMicrons": 35.0, "medianRadiusMicrons": 2.0, "sigmaMicrons": 2.0, "minAreaMicrons": 10.0, "maxAreaMicrons": 400.0, "threshold": 0.18, "maxBackground": 0.0, "watershedPostProcess": true, "excludeDAB": false, "cellExpansionMicrons": 5.0, "includeNuclei": true, "smoothBoundaries": true, "makeMeasurements": true, "thresholdCompartment": "Nucleus: DAB OD mean", "thresholdPositive1": 0.65, "thresholdPositive2": 0.4, "thresholdPositive3": 0.6, "singleThreshold": true}') |
| --- | --- |
| PIMO staining | runPlugin('qupath.imagej.detect.cells.PositiveCellDetection', '{"detectionImageBrightfield": "Hematoxylin OD", "requestedPixelSizeMicrons": 0.5, "backgroundRadiusMicrons": 35.0, "medianRadiusMicrons": 2.0, "sigmaMicrons": 2.0, "minAreaMicrons": 10.0, "maxAreaMicrons": 400.0, "threshold": 0.025, "maxBackground": 0.0, "watershedPostProcess": true, "excludeDAB": false, "cellExpansionMicrons": 5.0, "includeNuclei": true, "smoothBoundaries": true, "makeMeasurements": true, "thresholdCompartment": "Cytoplasm: DAB OD mean", "thresholdPositive1": 0.1, "thresholdPositive2": 0.4, "thresholdPositive3": 0.6, "singleThreshold": true}') |

**Hotspot detection settings**

The script provided on <https://gist.github.com/Svidro/6171d6d24a85539d3af5d417bc928d50#file-hotspot-detection-0-2-0m8-groovy> requires the definition of three parameters shown in the table below. The same settings were used for both markers.

| **Parameter** | **Definition** | **Settings** |
| --- | --- | --- |
| minCells | minimum number of cells in a hotspot | 10 |
| radiusMicrons | Distance between cells | 25 |
| pixelDensity | Changes with the other variables, requires testing | 1 |
